# The Pre-synaptic Landscape of Mitral/Tufted Cells of the Main Olfactory Bulb

**DOI:** 10.3389/fnana.2019.00058

**Published:** 2019-06-06

**Authors:** Amit Vinograd, Gen-ichi Tasaka, Lena Kreines, Yair Weiss, Adi Mizrahi

**Affiliations:** ^1^Department of Neurobiology, Alexander Silberman Institute of Life Sciences, The Hebrew University of Jerusalem, Jerusalem, Israel; ^2^The Edmond and Lily Safra Center for Brain Sciences, The Hebrew University of Jerusalem, Jerusalem, Israel

**Keywords:** olfaction, olfactory bulb, rabies tracing, mitral cells, neuronal circuits, top-down inputs

## Abstract

In olfaction, all volatile odor information is tunneled through the main olfactory bulb (OB). Odor information is then processed before it is transferred to higher brain centers. Odor processing in the OB is carried out by numerous local inhibitory circuits and modulated by top-down input. Top-down modulation of OB function has been shown to act *via* interneurons but evidence also exists for its direct impact onto the principle mitral and tufted cells (M/Ts). Here, we used monosynaptic rabies trans-synaptic tracing from the OB to map and quantify the local and top-down pre-synaptic landscape of M/Ts and local inhibitory interneurons. We found that M/Ts receive a significant amount of top-down inputs from various brain regions that match qualitatively but not quantitatively those that synapse onto local inhibitory inter-neurons. These results show that M/Ts are direct targets of top-down inputs.

## Introduction

The anatomical and cellular organization of the main olfactory bulb (OB) has been defined in detail (Shepherd, 1972; Wilson and Mainen, 2006; Adam and Mizrahi, 2010; Kosaka and Kosaka, 2016). To a large extent, the method of choice to study local connectivity patterns in the OB was dominated by slice Electrophysiology. However, synaptic mapping with electrophysiology can be limited, especially when the synaptically connected neurons are spatially distant. Electrophysiology is also limited in studying precise connectivity patterns from top-down projections. Studies of top-down connectivity into the OB have been carried out by classic tracing studies which label axonal projection patterns. Tracing studies suggest that top-down inputs make contacts predominantly onto inhibitory interneurons (INs; Shepherd, 1972; Gómez et al., 2005; Kiselycznyk et al., 2006; Wilson and Mainen, 2006; Matsutani and Yamamoto, 2008; Padmanabhan et al., 2019). As a result, models of OB function emphasize the role of top-down inputs primarily *via* INs (Gracia-Llanes et al., 2010b; Koulakov and Rinberg, 2011; Linster and Cleland, 2016; Yamada et al., 2017). Anatomy of axonal projections alone, however, does not explicitly reveal synaptic connectivity patterns. Here, we asked to what extent do top-down inputs also synapse on the mitral/tufted cells (M/Ts), the principal neurons of the OB.

Indirect evidence supports the notion of top-down inputs directly onto the M/Ts. Neurons outside the bulb are known to release unique neurotransmitters that have their receptors on the membrane of M/Ts. For example, the α4β2-like and the β2 nicotinic receptors are enriched in the mitral cell layer (MCL; Wada et al., 1989; Le Jeune et al., 1995); the 5-HT receptor 5HT2c was found to enable direct excitation of M/Ts (Hardy et al., 2005) and the 5-HT receptor 5HT2A and the noradrenergic β1 receptor were shown to be co-localized primarily on mitral cells (Yuan et al., 2003).

Top-down connectivity is thought to play important roles in the function of any neural circuit. In the OB, for example, top-down inputs from the piriform cortex (PCx) and anterior olfactory nucleus (AON) have been shown to increase the signal to noise ratio of odor inputs (Markopoulos et al., 2012; Otazu et al., 2015), synchronize granule cells activity (Manabe et al., 2011; Boyd et al., 2012) and coordinate odor inputs across bulbs (Grobman et al., 2018). Noradrenergic top-down inputs were shown to be important for odor discrimination learning (Doucette et al., 2011) and even memory formation (Shea et al., 2008). Therefore, mapping the direct bulbar targets of top-down inputs is an important initial step towards a mechanistic understanding of their function and the function of the OB at large.

Here, we used rabies virus tracing, a relatively new tool for studying connectivity, to examine the pre-synaptic landscapes onto genetically identified cell types in the OB—INs and M/Ts (Wickersham et al., 2007; Miyamichi et al., 2013). Using rabies, we examined both local and top-down pre-synaptic partners onto M/Ts and INs qualitatively and quantitatively.

## Materials and Methods

### Animals

We used Tbet-cre (Haddad et al., 2013) and GAD-cre (Taniguchi et al., 2011) females (10–16 weeks old, both on a background of a C57BL/6 strain). Animal care and experiments were approved by the Hebrew University Animal Care and Use Committee.

### DNA Constructs

pAAV-CAG-FLEx-oG was constructed using standard molecular cloning methods based on polymerase chain reaction (PCR) and restriction enzymes commercially available from New England Biolabs. Briefly, oG amplified by PCR from pAAV-EF1a-DIO-oG (Addgene Plasmid #74290; a gift from Edward Callaway; Kim et al., 2016) was subcloned into pAAV-CAG-FLEx-RG (Addgene Plasmid #48333; Miyamichi et al., 2013) digested with SalI and AscI. The avian tumor virus receptor A (TVA) plasmid pAAV-CAG-FLEx- TVA^66T^ was purchased from Addgene (Plasmid #48331; Miyamichi et al., 2013).

### Viral Procedures

EnvA-Pseudotyped RabiesΔG (RVΔG; 3 × 10^11^ infectious particles per ml) was produced following an established protocol (Wickersham et al., 2007; Osakada and Callaway, 2013). Adeno associated viral (AAV) vectors containing CAG-FLEx-TVA^66T^ (2 × 10^12^ genomic copies per ml), and CAG-FLEx-oG (10^12^ genomic copies per ml) were produced by the viral vector core of the Edmond and Lily Safra Center for Brain Sciences.

### Virus Injections

For trans-synaptic labeling, we anesthetized mice with an intraperitoneal injection of ketamine and medetomidine (100 and 0.83 mg/kg, respectively) and a subcutaneous injection of carprofen (0.004 mg/g). Two-hundred nanoliter of 1:2 mixture of AAV2 CAG-FLEx-TVA^66T^-mCherry and AAV2 CAG-FLEx-oG was injected into the dorsal surface of the OB, in either one, or two bulbs by using Nanoject (Drummond Scientific). Injections into Tbet-cre mice were targeted to 250–350 μm underneath the dura surface. Injections into GAD-cre mice were targeted to a depth of 350–650 μm. To ensure high and stable expression of the AAVs we waited at least 2 weeks before a 100 nl of RVΔG-GFP was injected into the same locations and depths.

### Histology

Five days after rabies injections, mice were sacrificed with an overdose of Pental and were perfused transcardially with phosphate-buffered saline (PBS) followed by 4% paraformaldehyde (PFA) in PBS. Brains were post-fixed for 12–24 h in 4% PFA in PBS and then cryoprotected for >24 h in 30% sucrose in PBS. Then, 60 μm coronal slices of the entire brain were made using a freezing microtome (Leica SM 2000R) and preserved in PBS. Prior to mounting on microscope slides, slices were incubated with DAPI (Santa Cruz Biotechnology; 50 μg/ml) for 5 min and then washed. Sections were imaged using an Olympus IX-81 epifluorescent microscope with a 4× and 10× objective lens (0.16 and 0.3 NA; Olympus) and a Leica SP-5 confocal microscope, using a 40× (1.3 NA) oil objective.

### Data Analysis

Images were analyzed using ImageJ. The brightness and contrast of each channel were manually adjusted. In cases where cell density was too high, several images in different focal planes were taken, and in few cases, slices were further imaged using a confocal microscope. To ensure robustness of the counting, images were counted twice, independently, by two different people. The counting was made in each channel separately using the sync windows and cell counter plugins in ImageJ. For reference atlases, we used the Allen brain atlas^1^.

## Results

### Mapping Pre-synaptic Landscapes Using Rabies Trans-synaptic Tracing

**Figure 1:**
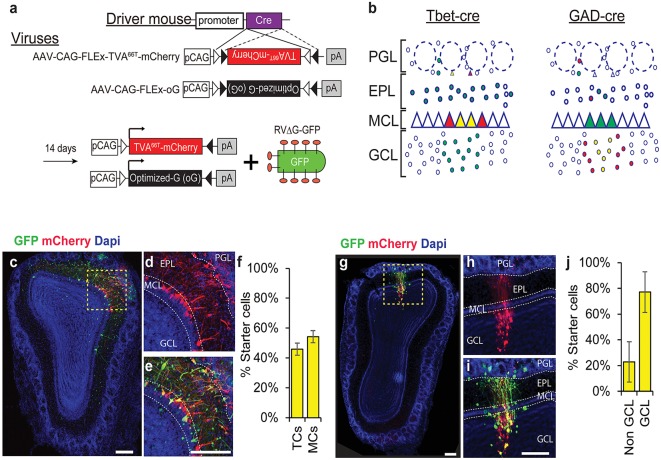
Rabies trans-synaptic tracing of mitral and tufted cells (M/Ts) and interneurons (INs) in the olfactory bulb (OB). **(A)** A schema of the adeno associated viral (AAV) vectors used in our experiments. The tumor virus receptor A (TVA)^66T^ and the envelope glycoprotein—oG are designed in a cre dependent manner (top). Injection of the AAV into a cre expressing mouse induces recombination and expression of TVA^66T^ and oG in a cell-specific manner. A pseudotyped rabies virus expressing GFP (RV-ΔG-GFP) is then injected to the same location in the OB to reveal the pre-synaptic landscape of the previously infected neurons. **(B)** Illustration of two different cre expressing driver mice and their expected result after injection of all the rabies components. Tbet-cre mice are expected to have starter cells (yellow) and mCherry (red) expressing neurons in M/Ts and their input cells should be green only (left). In GAD-cre mice, yellow and red cells should be restricted to INs (right). Only the local circuitry is shown for clarity. **(C)** Photomicrograph showing a coronal section of an OB slice after injecting the AAVs and RV into a Tbet-cre mouse. **(D)** Inset of the area marked in “**C**” showing the mCherry channel. Notice that mCherry expression is restricted to the MCL and juxta-glomerular area, indicating the specificity to M/Ts cells. **(E)** Magnified view of the inset in “**C**”. Yellow starter cells are detected as M/Ts cells. **(F)** Quantification of the number of starter cells in Tbet-cre mice, representing tufted cells (TC) and mitral cells (MCs) separately. **(G–J)** Same as “**C–F**” but for GAD-cre mice, where INs are now the starter cells. Scale bars −200 μm. PGL, periglomerular layer; EPL, external plexiform layer; MCL, mitral cell layer; GCL, granule cell layer.

To identify the pre-synaptic inputs of different subpopulation of neurons in the OB we used a cre-dependent monosynaptic rabies trans-synaptic tracing system. We used a mutated version of the TVA receptor (TVA^66T^) that allowed us to trace simultaneously both local and long range inputs with minimal noise levels (Miyamichi et al., 2013). In addition, we used the envelope optimized glycoprotein—oG (Kim et al., 2016). Cre-dependent TVA^66T^ and oG were packaged into AAVs and cell type specificity was achieved by the mouse driver line expressing Cre recombinase in specific subpopulations (Figure 1A).

We focused on two major classes of neurons—principal neurons (the M/Ts) and the local inhibitory interneurons (INs). For the M/T cells, we used the Tbet-cre mice, which express cre exclusively in M/Ts (Haddad et al., 2013), and collectively form ~1% of the total cell population in the OB (Shepherd, 1972; Urban and Arevian, 2009). Although M/Ts are few, they are the main output channel of this brain region. For tracing INs we used GAD-cre mice, which express cre in all inhibitory neurons (Taniguchi et al., 2011). INs are composed of different subpopulations but their output is restricted to the local circuitry, suggesting that they are mainly contributing to local computations (Shepherd, 1972; Parrish-Aungst et al., 2007).

To label inputs onto M/Ts and INs, we injected Tbet-cre and GAD-cre mice, respectively. We first injected AAVs (TVA^66T^-mCherry and oG), and then rabies (RVΔG-GFP) at least 14 days later. Five days following rabies injection, we sliced the brains and quantified the number and location of all labeled neurons across the brain. In Tbet-cre mice, only M/Ts expressed mCherry^+^ or mCherry^+^GFP^+^. These mCherry^+^GFP^+^ neurons are, thus, the “starter cells” from which tracing begins. Starter cells were located in the MCL (representing MCs; mean ± SEM: 54.1% ± 4%) and the superficial part of the external plexiform layer (EPL; representing tufted cells: mean ± SEM: 45.9% ± 4%) but not in the granule cell layer (GCL; Figures 1B–F). Thus, this system specifically targets projection neurons with minimal leakage to inhibitory neurons. Importantly, injecting rabies into OBs with no prior injection of AAV- TVA^66T^-mCherry virus, resulted in no GFP^+^ cells in the OB. The specificity to M/Ts was similar to that reported in our previous article using a different M/T-specific cre line (Pcdh-cre; Miyamichi et al., 2013).

Injecting the components of the rabies system into GAD-cre mice resulted in starter cells that are specific to the local inhibitory neurons (Figures 1B,G–J). In these mice, starter cells were identified predominantly in the GCL (77.1% ± 16%; Figures 1G–J). In both mouse lines, we never identified cells expressing mCherry outside the main OB.

### The Pre-synaptic Landscape of M/Ts

**Figure 2:**
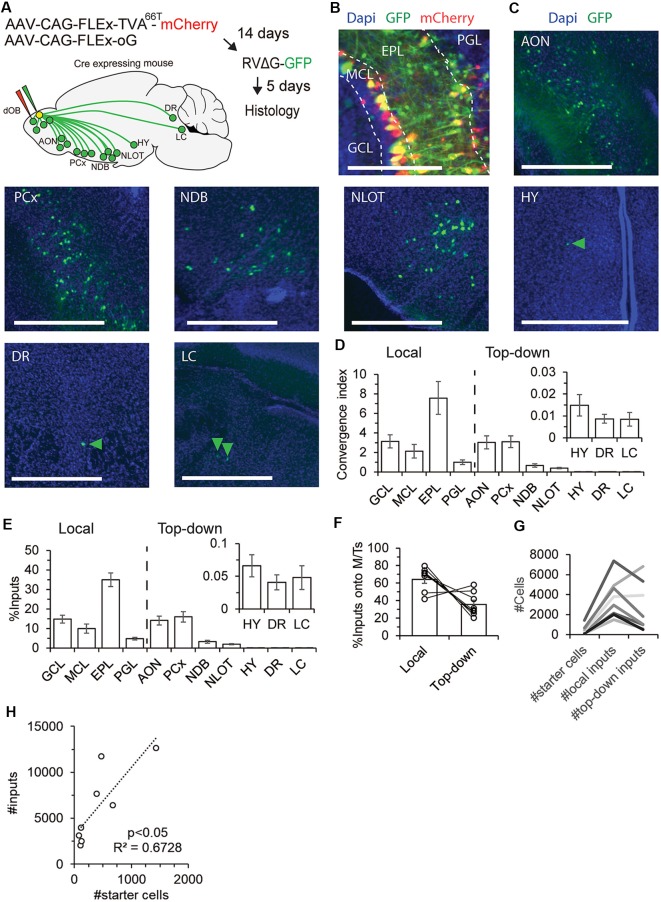
The pre-synaptic landscape of M/Ts. **(A)** An illustration of the experimental paradigm of injections and resultant input map from the main sources of inputs to the OB. Input cells were identified in the OB, anterior olfactory nucleus (AON), piriform cortex (PCx), nucleus of the diagonal band area (NDB), nucleus of the lateral olfactory tract area (NLOT), hypothalamus area (HY), dorsal raphe (DR) and locus coeruleus (LC). A similar map was revealed for both M/Ts and INs (see Figure 3). **(B)** Representative image of starter cells and local inputs from different layers of the OB. Scale bar – 200 μm. **(C)** Representative examples of top-down inputs onto M/Ts from seven of the different brain regions containing GFP^+^ signal. Arrowheads point to sparse GFP labeled neurons. Scale bars −500 μm. **(D,E)** Distribution of the convergence index (CI; **D**) and percentage **(E)** of inputs onto M/Ts, by brain region. Locally, neurons from four layers send inputs to M/T cells. CI from seven brain regions sending top-down input onto M/Ts (other regions were not quantified given their sparser representation in the data). **(F)** Bar graphs showing the percentage of local and top-down inputs onto M/Ts per mouse. **(G)** Graph comparing the ratio between the number of starter cells and local and top-down input cells in individual mice (each line is a single mouse). Higher number of starter cells resulted with more inputs. In 6/8 mice the majority of inputs were local. **(H)** Total input cells relative to the number of starter cells. Each point is a single mouse. The dotted line is a linear fit.

To map the local and top-down inputs onto M/Ts, we analyzed GFP^+^ neurons inside and outside of the OB in Tbet-cre injected mice (Figure 2; *N* = 8 mice). To quantify the inputs from each brain region, we used an index called convergence index (CI), which is the number of pre-synaptic GFP^+^ input cells from a given region divided by the number of starter cells. In addition, we also calculated the percentage of inputs from each brain region, a value that does not depend on the number of starter cells.

First, we quantified the inputs from local neurons in the OB. To do so, we scored CI in four regions of the OB—periglomerular layer (PGL), EPL, GCL, and the MCL (Figure 2B). Input neurons from the EPL are most likely parvalbumin-positive neurons (Miyamichi et al., 2013). Inputs from the PGL are potentially from a diverse set of INs, and inputs from the GCL are likely granule cells. The inputs from the MCL were mostly small-somata unidentified neurons but a few inputs were also large-somata MCs. Thus, inputs from the “MCL” can include granule cells, deep short axon cells (dSAC), or MCs (Figure 1C). Consistent with our previous findings, most inputs to M/Ts arise from the EPL (Figure 2D; CI = 7.6 ± 1.7 in the EPL, compared to 3.1 ± 0.7 in the GCL; Miyamichi et al., 2013).

Second, we quantified the CI from brain regions outside the OB (Figures 2C,D). GFP-labeled neurons were found in the AON (8/8 mice), PCx (8/8 mice), nucleus of the diagonal band area (8/8 mice), nucleus of the lateral olfactory tract area (8/8 mice), hypothalamic regions (6/8 mice), dorsal raphe (DR; 7/8 mice) and locus coeruleus (LC; 7/8 mice). In 50% of the mice, few neurons were also identified in the CA1 of the hippocampus (4/8 mice) and in the entorhinal cortex (4/8 mice, see also (Padmanabhan et al., 2019); data not shown). The highest numbers of neurons sending input to M/Ts were identified in the AON and PCx (Figure 2D; CI = 3.0 ± 0.7 in AON; 3.2 ± 0.6 in PCx). These values were qualitatively similar when examining the percentage instead of the CI (Figure 2E). M/Ts received considerably more local than top-down inputs: ~70% of the total inputs were local (Figure 2F), and 6/8 mice had more local inputs than top-down inputs regardless of the number of starter cells (Figure 2G). Importantly, the number of total inputs increased linearly with the number of starter cells (Figure 2H). Thus, our results do not suffer from sublinear effects due to different numbers of starter cells in different mice. Taken together, these results show that although M/Ts do receive most of their inputs from local neurons, significant levels (~30%) of direct monosynaptic inputs arise from neurons outside the OB. A schema of the brain depicting the major sources of inputs onto M/Ts is shown in Figure 2A.

### The Pre-synaptic Landscape of Inhibitory Interneurons

**Figure 3:**
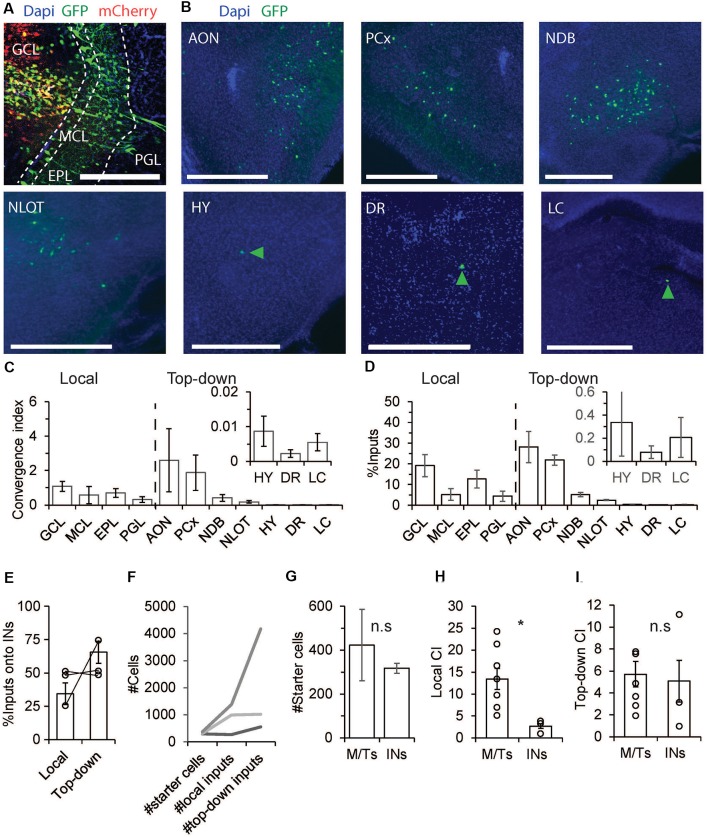
The pre-synaptic landscape of INs. **(A–F)** Same as Figures 2B–G but for GAD-cre mice. Unlike M/Ts, INs have more inputs from top-down sources. **(G)** M/Ts and INs had similar number of starter cells (*p* > 0.05, Wilcoxon rank-sum test). **(H)** INs had significantly lower CI levels from local inputs as compared to M/Ts (*p* < 0.05 Wilcoxon rank-sum test). **(I)** INs and M/Ts receive comparable inputs from top down brain regions (*p* > 0.05, Wilcoxon rank-sum test). *p < 0.05, n.s not significant.

Next, and in order to have a reference to the M/Ts data, we repeated the same experiments mentioned above in GAD-cre mice (*N* = 3 mice). Qualitatively, we found that local and top-down inputs from all regions that were found as inputs to M/Ts were found in the GAD-cre mice as well (Figures 3A–D; compare Figures 2D,E, 3C,D). However, as expected, there were clear quantitative differences. The local inputs to INs were not biased towards the EPL as in the M/Ts input landscape. On the contrary, inputs came equally from all layers of the OB (Figures 3C,D). The pattern of inputs to interneurons is complex as interneurons are a heterogeneous group of cell types. Here, we lack cell-type specificity of both the starter cells and the input cells and any combination of connectivity remains possible. For example, input neurons from the GCL probably reflect dSAC as dSAC have been shown to make synaptic contacts selectively on GABAergic INs (Gracia-Llanes et al., 2003; Eyre et al., 2008; Kosaka and Kosaka, 2011). Other combinations of interneuron-interneuron connectivity can be revealed by combining cell-type specific markers together with rabies tracing.

Unlike the pre-synaptic landscape of M/Ts, the input to INs was mostly from outside the OB (Figures 3E,F). The highest CI levels onto INs were found from the PCx and AON (Figure 3C; AON: CI = 2.6 ± 1.8, PCx: CI = 1.9 ± 1). On average, INs and M/Ts show similar numbers of starter cells (Figure 3G). However, while INs had significantly less CI values from local inputs as compared to M/Ts (Figure 3H, INs: 2.7 ± 0.5, M/Ts: 13.5 ± 2.4, *p* < 0.05, Wilcoxon rank-sum test), both groups had similar CI values from top-down inputs (Figure 3I; M/Ts: 5.7 ± 1.2, INs: 5.1 ± 1.9, *p* > 0.05, Wilcoxon rank-sum test). Taken together, these results show that while M/Ts have relatively more local inputs, top-down inputs as a whole target rather equally M/Ts and INs.

## Discussion

We used monosynaptic rabies trans-synaptic tracing to quantify the inputs onto M/Ts in the main OB. Using this approach, we provide a quantitative and qualitative evaluation of the distributions of top-down and local inputs as detected with rabies. We complement this analysis by comparing the landscape of M/Ts to those of local INs. Our results suggest that M/Ts have a significant amount of direct inputs from long range sources.

### Rabies Tracing in the OB—Pros and Cons

The OB is an anatomically well-documented brain structure. Its organization in terms of local and long-range connectivity was revealed by histological and electrophysiological profiling, as well as by various anatomical methods (Shepherd, 1972; Egger and Urban, 2006; Wilson and Mainen, 2006; Kosaka and Kosaka, 2016). Monosynaptic rabies virus tracing, a relatively recent technique, adds another layer of information to our understanding of OB connectivity (Miyamichi et al., 2013; Kohl et al., 2018).

Rabies tracing is used to study synaptic connectivity patterns, *in vivo*. It has several strengths. Given its genetic nature, it allows for synaptic tracing with good cell-type specificity (Wickersham and Feinberg, 2012; Callaway and Luo, 2015; Luo et al., 2018). As a result, we were able to trace connectivity from a relatively sparse neuronal sub-types like the M/Ts, which are ~1% of the total neuronal population in the OB (Shepherd, 1972; Urban and Arevian, 2009). Rabies tracing also allows quantification of the input landscape, from the exact same cells, all at once. This allowed us to compare the level and distribution of inputs onto M/Ts and INs. A particular strength of rabies tracing is its ability to label long range inputs, which can be centimeters away (Callaway and Luo, 2015; Luo et al., 2018). Finally, the recent improvements made on decreasing noise levels allow simultaneous tracing of both local and long-range connectivity (Miyamichi et al., 2013).

Rabies tracing has limitations that are important to keep in mind. A major limitation is the low efficiency in terms of revealing the full breadth of synapses on a given cell type. While false positives are rare, false negatives are common. Another caveat is that rabies tracing might not label all pre-synaptic cells with the same probability (Callaway and Luo, 2015). Thus, our data is by no means a reference to the absolute numbers of pre-synaptic partners each cell type receives. More recent versions of the rabies system (Chatterjee et al., 2018), or combining more than one approach will yield better estimates in future work (Luo et al., 2018; Lo et al., 2019). In addition, physiological properties of synapses, beyond contact, are not reflected by rabies tracing (Callaway and Luo, 2015). In fact, it was recently shown that the quality of tracing does not correlate with recent activity of the synapses traced (Beier et al., 2017); let alone their synaptic strength. Therefore, a more complete picture of the functional implications of connectivity would arise by combining anatomical measurements with functional ones.

Keeping its caveats and strengths in mind, rabies tracing allowed us to reveal a large number of the known inputs to the OB—i.e., a total of at least nine brain regions that provide top-down inputs onto the OB (Shepherd, 1972; Gómez et al., 2005; Kiselycznyk et al., 2006; Wilson and Mainen, 2006; Padmanabhan et al., 2019). This result also suggests that the majority of cell -types are already represented by the Tbet-cre and GAD-cre mice and that the rabies system faithfully represents the classical known inputs onto the OB (Shipley and Adamek, 1984). Given that we can find a quantitative difference between the pre-synaptic landscapes of excitatory and inhibitory neurons, further dissection is needed. INs alone are composed of more than a dozen cell-types and their unique input landscape are not fully resolved (Tepe et al., 2018). M/Ts are, at the very least representing two different subtypes, which are known to be physiologically distinct (Adam et al., 2014), and their unique input landscape is also unknown. With advances in scRNA sequencing and our increasing ability to define cell types (Zeng and Sanes, 2017), the specificity of rabies tracing will become even more evident and can be used as an additional tool to map the pre-synaptic landscape onto different neuronal subtypes in the OB with greater specificity.

### The Pre-synaptic Landscape of the Main OB

Local INs in the OB are considered to have major roles in the processing of odor information. These can include computations like contrast enhancement, gain control, history-dependent processing, and decorrelation of odor inputs (Wilson and Mainen, 2006; Kato et al., 2012; Cleland, 2014; Gschwend et al., 2015; Vinograd et al., 2017b). Many of these computations are known to be influenced by top-down inputs. For example, AON to granule cell connectivity facilitate mitral cell activity to increase the signal to noise levels of their output (Oettl et al., 2016). Similarly, neuromodulatory inputs such as noradrenaline, acetyl-choline and serotonin are considered to act on M/Ts activity through local INs (Wilson and Mainen, 2006; Gracia-Llanes et al., 2010a; Linster and Cleland, 2016). Recent studies, however, indicate a possible influence of top-down inputs onto M/Ts activity directly. Markopoulos et al. (2012) examined functional properties of projections from the AON to the OB and found that these directly excite MCs. Kapoor et al. (2016) showed that optogenetic excitation of raphe nucleus fibers in the OB leads to a fast modulation of M/Ts activity, and Gracia-Llanes et al. (2010a) used a combination of immunohistochemistry with electron microscopy showing that serotonergic inputs from the raphe nuclei make synaptic contacts onto principal cells in the OB. Here we show that INs and M/Ts receive inputs from the exact same top-down regions. What could be the function of similar inputs that impinge upon both excitatory M/Ts and INs? One hypothesis is that top-down inputs are not selective and impact the circuit as a whole, determining state-like configurations. Another hypothesis is that the top-down inputs are actually locally differential. Different odor channels may be targeted differently based on factors such as experience. For example, top-down inputs can target specific M/Ts and inhibit others *via* INs. We recently showed that mitral cell responses to odors can be selectively inhibited or strengthened following specific experience (Vinograd et al., 2017a). Since top-down inputs have direct access to both increase or decrease specific output channels of odor information, they could serve as a mechanism to determine tuning specificity of OB output. Lastly, the analysis of neuronal inputs used here was based on anatomical location alone. Future studies that will combine rabies tracing with other techniques like *in situ* hybridization or immunohistochemistry will add another layer of information describing input neurons to M/T cells.

## Data Availability

The raw data supporting the conclusions of this manuscript will be made available by the authors, without undue reservation, to any qualified researcher.

## Ethics Statement

Animal care and experiments were approved by the Hebrew University Animal Care and Use Committee.

## Author Contributions

AV and AM designed the experiments and wrote the article. YW and LK analyzed data. GT conducted experiments. AV conducted experiments and analyzed the data.

## Conflict of Interest Statement

The authors declare that the research was conducted in the absence of any commercial or financial relationships that could be construed as a potential conflict of interest.

## Footnotes

^1^http://atlas.brain-map.org
